# P01-021 – Macrophage migration inhibitory factory in FMF

**DOI:** 10.1186/1546-0096-11-S1-A25

**Published:** 2013-11-08

**Authors:** I Sari, Y Savran, DL Kozaci, N Gunay, F Onen, S Akar

**Affiliations:** 1 Rheumatology, Dokuz Eylul University School of Medicine, Izmir, Turkey; 2 Internal Medicine, Dokuz Eylul University School of Medicine, Izmir, Turkey; 3 Biochemistry, Adnan Menderes University School of Medicine, Aydin, Turkey; 4 Biochemistry, Adnan Menderes University School of Medicine, Izmir, Turkey

## Introduction

Familial Mediterranean Fever (FMF) is an autoinflammatory disorder characterized by recurrent, inflammatory, self-limited episodes of fever and serositis. Neutrophils are one of the key players in the pathophysiology of FMF. Macrophage migration inhibitory factor (MIF) is a pleiotropic cytokine involved in several inflammatory processes including innate and adaptive immune responses . In addition, MIF has been shown to regulate trafficking of inflammatory cells includingneutrophis to the sites of inflammation. Because its association with innate immunity, leukocyte trafficking, and inflammation MIF may be considered as an attractive cytokine in the pathogenesis of FMF.

## Objectives

In this study we aimed to investigate MIF levels and its relationship with M694V mutations in patients with FMF.

## Methods

Fifty one unrelated attack free FMF patients (14 M and 27 F, 32.8±8.7 years) and 30 healthy controls (16 M and 14 F, 32.7±7 years) were included in the study. Serum MIF were studied and allele frequency of M694V was calculated.

## Results

Age, sex distribution, waist circumference, body mass index, smoking status and serum lipids were not different between the patient and control groups (P > 0.05). On the other hand, the levels of CRP, ESR, and MIF were significantly higher in FMF patients compared to those of controls (P < 0.05; 4.7±7.1 vs. 1.8±2 mg/L, 15.8±17 vs. 8.3± 5.2 mm/h, and 30.1±18.8 vs. 9±4.4 ng/mL respectively). Comparison of patients with and without M694V mutation revealed that MIF levels were not different between the groups. Regression analysis showed that none of the variables including disease duration, CRP, ESR, and BMI were predicting MIF concentrations (P > 0.05).

## Conclusion

In this study we showed that: (1) MIF concentrations were significantly higher in attack-free FMF patients compared to healthy subjects; (2) increased MIF levels were independent from the inflammatory activity as assessed by ESR and CRP and (3) M694V mutations had no impact on MIF concentrations.

## Disclosure of interest

None declared.

